# Electrophysiological Characterization of the Antarease Metalloprotease from *Tityus serrulatus* Venom

**DOI:** 10.3390/toxins9030081

**Published:** 2017-02-27

**Authors:** Irene Zornetta, Michele Scorzeto, Pablo Victor Mendes Dos Reis, Maria E. De Lima, Cesare Montecucco, Aram Megighian, Ornella Rossetto

**Affiliations:** 1 Dipartimento di Scienze Biomediche and Istituto CNR di Neuroscienze, Università di Padova, Via Ugo Bassi 58/B, 35121 Padova, Italy; irenezornetta@gmail.com (I.Z.); scorzetomichele@gmail.com (M.S.); cesare.montecucco@gmail.com (C.M.); 2 Laboratório de Venenos e Toxinas Animais, Departamento de Bioquímica e Imunologia, Instituto de Ciências Biológicas, Universidade Federal de Minas Gerais, Belo Horizonte 31270-901, MG, Brazil; reispvm@gmail.com (P.V.M.D.R.); lima.mariaelena@gmail.com (M.E.D.L.)

**Keywords:** antarease, scorpion, Zn-metalloprotease, vesicle associated membrane protein 2 (VAMP2), botulinum neurotoxins

## Abstract

Scorpions are among the oldest venomous living organisms and the family *Buthidae* is the largest and most medically relevant one. Scorpion venoms include many toxic peptides, but recently, a metalloprotease from *Tityus serrulatus* called antarease was reported to be capable of cleaving VAMP2, a protein involved in the neuroparalytic syndromes of tetanus and botulism. We have produced antarease and an inactive metalloprotease mutant in a recombinant form and analyzed their enzymatic activity on recombinant VAMP2 in vitro and on mammalian and insect neuromuscular junction. The purified recombinant antarease paralyzed the neuromuscular junctions of mice and of *Drosophila melanogaster* whilst the mutant was inactive. We were unable to demonstrate any cleavage of VAMP2 under conditions which leads to VAMP proteolysis by botulinum neurotoxin type B. Antarease caused a reduced release probability, mainly due to defects upstream of the synaptic vesicles fusion process. Paired pulse experiments indicate that antarease might proteolytically inactivate a voltage-gated calcium channel.

## 1. Introduction

Scorpions are widely diffused and have colonized a variety of environments, including very extreme ones and different urban areas [1,2]. They are predators acting via injection of powerful venoms that immobilize their prey. The *Buthidae* and *Hemiscorpiidae* scorpion families include the species dangerous for humans and their venoms are important human pathogens. Indeed, it has been estimated that scorpions are responsible for more than 1.2 million stings per year with almost 3000 deaths worldwide per year [1]. Scorpion human envenomation was officially designated as a neglected public health issue in the 2007 by the World Health Organization [3]. In Brazil, the number of scorpion accidents, reported in 2013 and 2014, was larger than the sum of accidents caused by bees, snakes, and spiders combined [4,5].

The effects of scorpion sting range from redness and pain to very severe and lethal effects, which result from failure of multiple organs eventually leading to death [1,6]. The range of pathological targets of scorpion venoms within the human body results from their complex composition that includes a variety of toxins (channel blocking neurotoxins, cardiotoxins, nephrotoxins, and hemolytic toxins) and enzymes (phosphodiesterases, phospholipases, hyaluronidases, and metalloproteinase) [7,8,9,10] and other active peptides, as antihypertensives [11]. The most widely studied components of scorpion venom are the ion channel-modulating toxins, which have been instrumental in the understanding of the structure-function relationship of many ion channels and of their role in neuronal physiology [12]. Among them are many insect toxins (for a review see: [13,14]). Although scorpion venom research has focused primarily on neurotoxic peptides, proteolytic activity has also been described [8,9,10]. Two types of proteases have been characterized in scorpion venom glands: serine proteases (SPSVs) [15] and metalloproteases [9,10]. Recently, a novel metalloprotease, called antarease, was isolated from the venom of *Tityus serrulatus.* This metalloprotease was reported to cleave VAMP2 close to the transmembrane domain [16]. VAMP2, together with the other two SNARE proteins termed SNAP25 and syntaxin, is essential for the release via exocytosis of a variety of biologically active molecules (neurotransmitters, hormones, peptides, and proteins) [17]. Fletcher et al. [16] have provided data indicating that antarease blocks exocytosis in pancreas and proposed that the VAMP2 cleavage activity is responsible for the well-known pancreatitis caused by scorpion stings [16,18]. More recently, antarease was suggested to be the first member of a novel class of scorpion metalloprotease toxins present in several scorpion genera [19].

The specific cleavage of VAMP2 was reported before to be at the basis of the neuroparalytic action of tetanus neurotoxin and botulinum neurotoxin types B, D, F, and G [20,21,22,23]. Therefore, we have decided to study the antarease action at the neuromuscular junction of mice and of *Drosophila melanogaster* third instar larvae using recombinant antarease and an active site antarease mutant, which should be devoid of any metalloprotease activity. We found that recombinant antarease is biologically active as it reduces neurotransmitter release both in invertebrate and vertebrate NMJs in a metalloprotease-dependent mode. However, we were unable to detect any VAMP2 cleavage under conditions in which botulinum neurotoxin type B proteolyzes its substrate.

## 2. Results

### 2.1. Purification of Recombinant Antarease and Its Mutant

The antarease complete sequence [19] was cloned in recombinant *E. coli* expression system, introducing an *N*-terminal c-Myc tag to facilitate its detection. Metalloproteases are characterized by the active site HExxH motif and it is established that the replacement of the Glu residue of the motif with Ala abolishes the enzymatic activity [24]. Therefore, we produced the E162A antarease mutant to be used as a control. 

**Figure 1:**
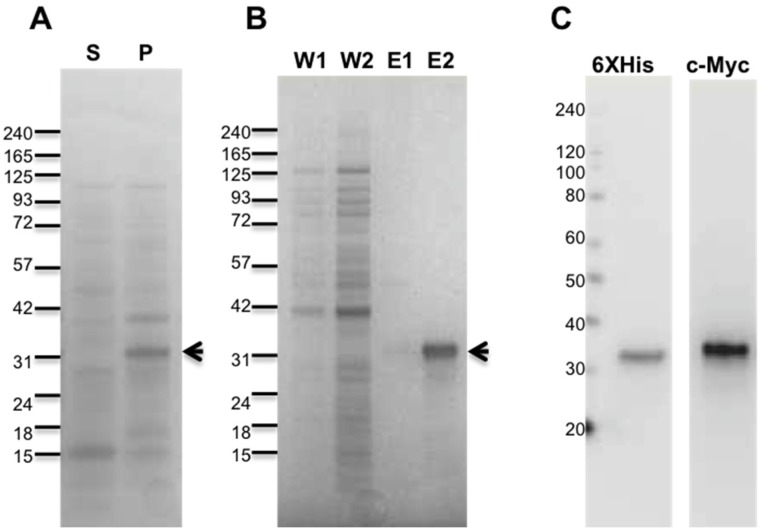
Purification and characterization of recombinant anterase. *T.* *serrulatus* antarease was produced as recombinant protein in *E. coli*. (**A**) SDS-PAGE showing the protein (arrow) association with the inclusion bodies (P), after bacterial cells lysis; (**B**) The pellet was extracted using mild solubilization. The recovered material was subjected to affinity chromatography. In the SDS-PAGE, washes (W1, W2) and eluted fractions (E1, E2) were shown. The fraction E2 containing antarease (arrow) was dialyzed over-night; (**C**) Antarease was characterized in immunoblotting, using specific antibodies against the *N*-terminal tags 6X-His-tag and c-Myc tag. The same purification protocol, yield, and purity were obtained for the E162A antarease mutant.

The two proteins were collected in the insoluble portion of the cells (Figure 1A and Figure S2), but 1% n-lauroyl sarcosine, a zwitterionic detergent, was found to be very effective in solubilizing the proteins. Recombinant antarease and its active site mutant were >95% pure as assessed by Coomassie Blue staining (Figure 1B and Figure S2). The expression and purification protocol used here yielded 1 mg pure protein per gram of cell paste. The identity of the proteins was confirmed by immunoblotting using specific antibodies for its *N*-terminal tags (Figure 1C). No further structural characterization was performed since a three-dimensional structure of antarease, to be used as reference, is not yet available. In addition, the recombinant antarease was found to be biologically active on insect and mammal neuromuscular junctions (see below).

### 2.2. Antarease, but Not Its Metalloprotease Active Site Mutant, Paralyzes the *Drosophila melanogaster* Neuromuscular Junction

The preys of scorpions range from insects to small reptiles and mammals, which are rapidly paralyzed by injected venom that spreads within the stung animal [25]. Therefore, we tested the effect of recombinant antarease and its E162A mutant on the evoked and spontaneous neurotransmitter release occurring at the *Drosophila melanogaster* neuromuscular junction (NMJ).

**Figure 2:**
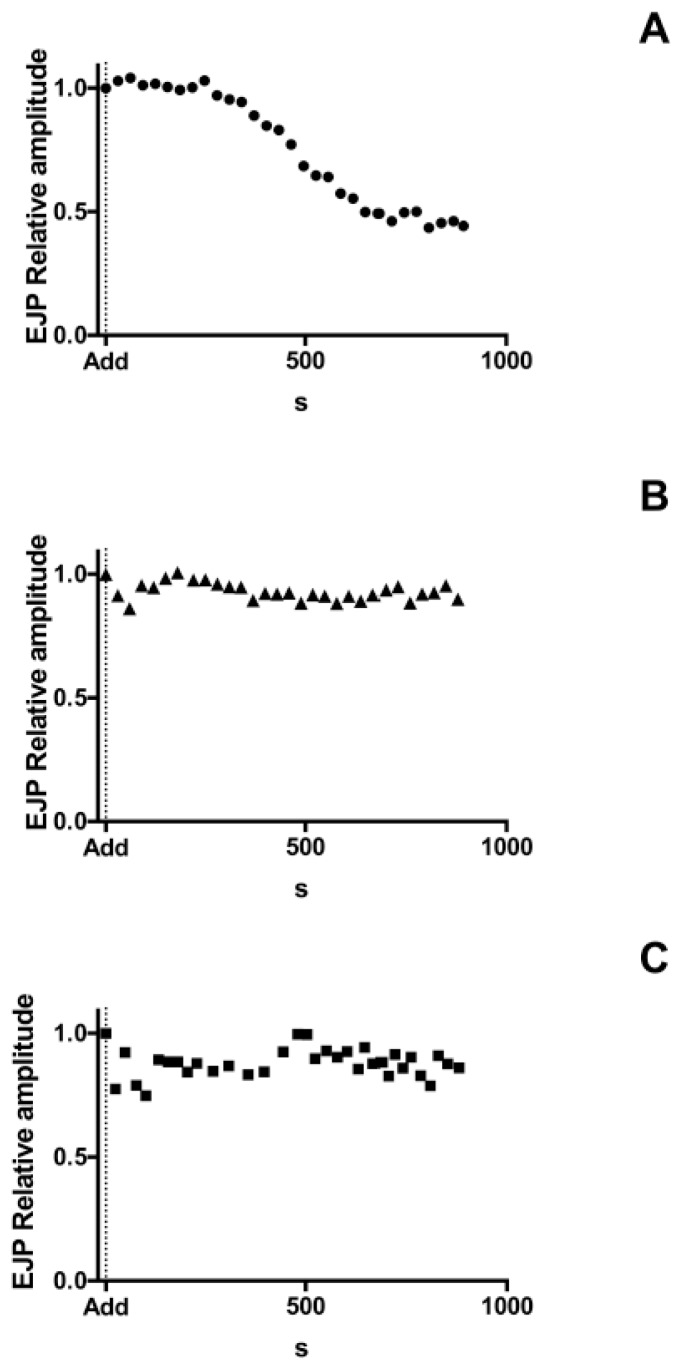
Time course of EJP amplitude in three example experiments following administration of Antarease, or antareaseE162A mutant, or vehicle alone. Antarease (**A**); antareaseE162A mutant (**B**); or vehicle alone (**C**) were added to the bath at “Add” (vertical dotted line). During the entire experiment, a microelectrode was intracellularly maintained in the same fibre and resting membrane potential was carefully controlled. Fibres showing a reduction in resting membrane potential below −60 mV, were discarded. For comparison, EJPs relative amplitudes are shown. The relative amplitude of each EJP was calculated with respect to the first recorded EJP in the experiment. Each experiment is representative of four runs per condition. Mean ± Standard Deviation of EJP Amplitudes of four experiments for each experimental condition, measured 500 s after addition of recombinant proteins or vehicle to the bath, are shown in Figure S3.

After addition to antarease or the mutant E162A or the vehicle alone to the bath, the amplitude of evoked Excitatory Junctional Potentials (EJPs) were recorded for 900 s in the same fiber clamped at −70 mV. EJPs were evoked at 0.1 Hz in order to prevent NMJ fatigue. Antarease caused a drastic decrease in EJPs at the *D. melanogaster* larvae NMJ. The effect of antarease was rather rapid as its amplitude declined within 10 min with a negligible response remaining during the observation period (Figure 2A). On the contrary, the antarease mutant E162A caused no effect (Figure 2B), similarly to the vehicle (Figure 2C). The rapidity of action of antarease together with the lack of toxic activity of its active site mutant indicate that antarease cleaves, via a metalloproteolytic activity, a substrate present on the external surface of the presynaptic membrane, which is essential for neurotransmitter release. On the basis of the rapidity of the effect, an action of antarease inside the nerve terminal, as it is the case of tetanus and botulinum neurotoxins [23], is very unlikely because there appears to be no sufficient time for antarease endocytosis and membrane translocation into the cytosol where its putative target VAMP2 is located.

### 2.3. Antarease, but Not Its Metalloprotease Active Site Mutant, Paralyzes Mouse Neuromuscular Junction

**Figure 3:**
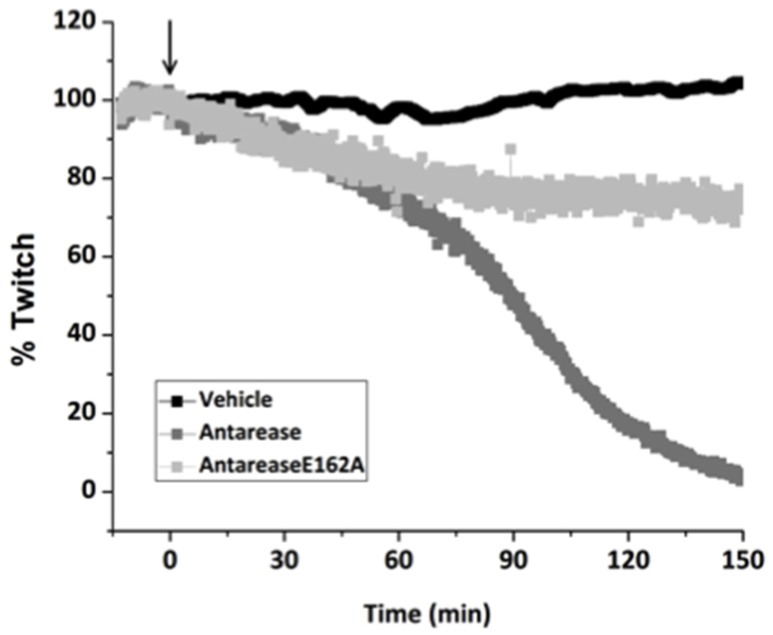
Antarease paralyzed the mouse phrenic nerve-hemidiaphragm preparation. Antarease (dark grey), antarease E162A (light gray), or vehicle (black) were added to the nerve-muscle preparations in Krebs-Ringer solution at 37 °C. Muscle twitch was induced by nerve stimulation and paralysis times were monitored. This experiment is representative of at least three runs per condition.

As the diet of *T. serratulus* may include small vertebrates, we tested the activity of antarease and of its E162A mutant on the mouse phrenic nerve-diaphragm NMJ preparation. This is a well-characterized test system for the neuroparalytic activity of presynaptic neurotoxins [26,27]. Figure 3 shows that antarease paralyses this mouse NMJ preparation with a t_1/2_ of about 90 min, whilst the mutant has a little effect. The reduction of muscle twitch capability was observed by indirectly stimulating muscle, whose twitch was rescued by direct muscle stimulation. This is a clear sign that antarease did not act on the muscle itself.

In contrast with botulinum neurotoxin [26,27], no lag phase was present here and the trace begun to run down right after toxin addition. This suggests that antarease acts proteolytically on a protein of the presynaptic membrane surface that is essential for neuroexocytosis. The slight effect of the inactive mutant can be explained by a slight inhibitory effect of its binding to the antarease target not followed by detachment because proteolysis does not take place. In simpler words, the mere binding of the mutant to the protein target of antarease could impair its function. Taken together, these experiments indicate that the antarease target is conserved from insects to mammalians.

### 2.4. Antarease Does Not Cleave the SNARE Proteins Involved in Neuroexocytosis

**Figure 4:**
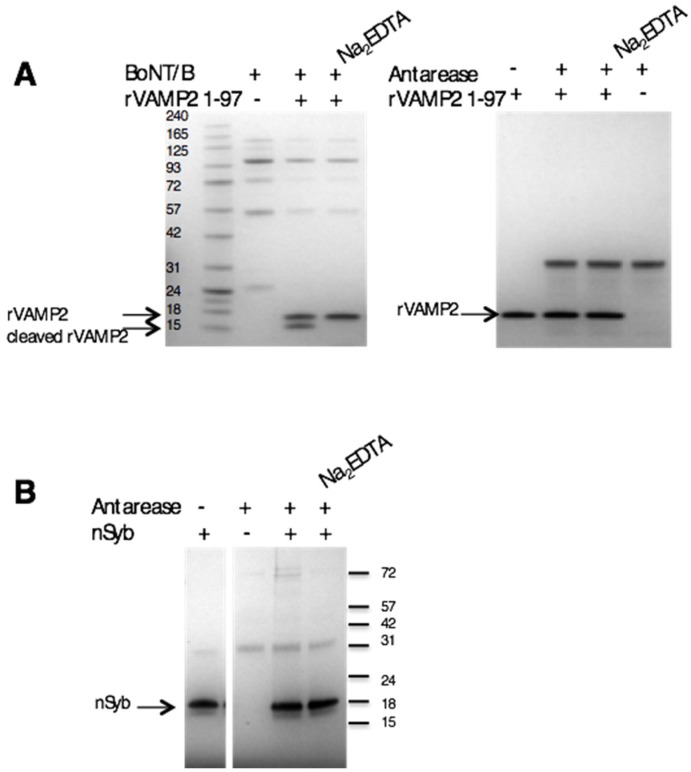
Cleavage assay of recombinant rVAMP2 and n-Syb. (**A**) The cytosolic domain of rat VAMP2 (segment 1–97) was not cleaved by antarease in our in vitro assay (right panel). BoNT/B proteolyzed the recombinant substrate and no cleavage was detected in the presence of Na_2_EDTA, which inhibits metalloproteases by chelating the active site zinc ion (left panel); (**B**) Drosophila simulans nSyb was produced in *E. coli* and used as substrate to test the proteolytic activity of antarease. It is clear that nSyb is not cleaved by antarease in our in vitro assay.

The three SNARE proteins VAMP, SNAP-25, and syntaxin are the core of the nanomachine that mediates the release of neurotransmitter, and, more in general, exocytosis [28,29]. Their cleavage by the metalloproteolytic activity of tetanus and botulinum neurotoxins leads to neuroparalysis [23]. Fletcher et al. (2010) [16] reported that antarease cleaves VAMP2 and suggested that this activity could be responsible for the pancreatitis that had been described following *T. serratulus* venom injection [19]. We have tested the metalloproteolytic activity of antarease on recombinant VAMP2 in vitro and have not been able to detect any proteolysis under conditions in which VAMP2 is cleaved by the botulinum neurotoxin type B (Figure 4A). To further analyze this aspect we incubated antarease with the *D. melanogaster* VAMP/synaptobrevin (n-Syb) and again we were unable to detect any cleavage (Figure 4B). This lack of enzymatic activity of recombinant antarease cannot be attributed to denaturation of the protease preparation or to a lack of active site Zn^2+^ because this very protein is biologically active on different neuronal biological preparations, as shown above.

### 2.5. Antarease Alters the Pair-Pulse Stimulation of the Drosophila melanogaster NMJ

**Figure 5:**
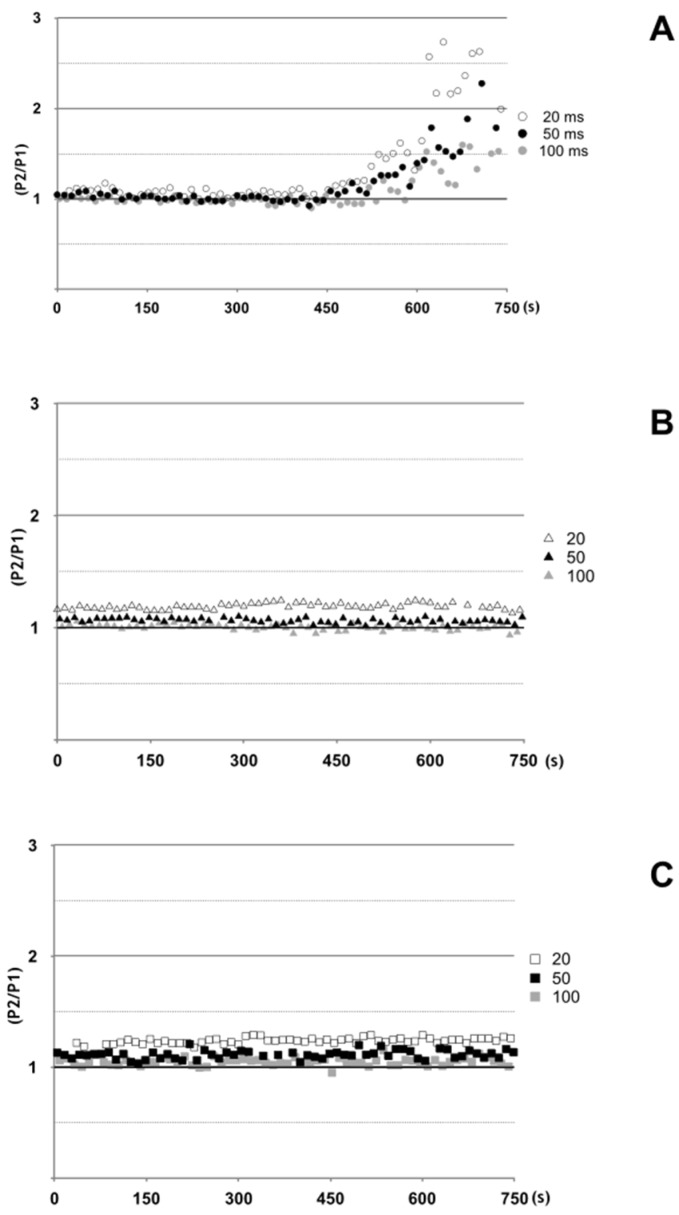
Time course of paired pulse facilitation in three example experiments following administration of antarease, antareaseE162A mutant, or vehicle alone. Antarease (**A**), antareaseE162A mutant (**B**), or vehicle (**C**) were added to the bath at time 0. During the entire experiment, microelectrode was intracellularly maintained in the same fiber. Resting membrane potential was carefully controlled during the entire experiment. Fibers showing a reduction in resting membrane potential below −60 mV, were discarded. Paired pulses were delivered with 20–50–100 ms delay between pulse 1 and pulse 2 for each experimental point (see methods). Mean and standard deviation of four experiments for each experimental condition, measured at 650 s after addition of recombinant proteins or vehicle are shown in Figure S4.

With the aim of a better definition of the metalloprotease dependent neuroparalytic action of antarease, we used the *D. melanogaster* larval NMJ which appeared to be a better target than the mouse NMJ in terms of rapidity of action. We performed a paired-pulse experiment, which measures the increase in synaptic response of a pulse delivered after a previous one at a defined time interval (Figure S1). Figure 5 shows that paired pulses stimulation induces a strong facilitation of the second response following the antaerase-depressed first EJP, in the third instar larval NMJ preparation (Figure 5). This ‘rescue’ effect is achieved very rapidly and is likely to involve the increase of presynaptic calcium responsible for paired-pulse facilitation. Taken together, the effect on single EJPs and the quick recovery of the second EJP in paired-pulse facilitation protocol do not support a complex mechanism of action of antarease similar to that of tetanus and botulinum neurotoxins which involves binding to the presynaptic membrane followed by endocytosis and membrane translocation into the cytosol [23].

## 3. Discussion

The main result presented here is that the *Tityus serrulatus* recombinant antarease causes the neuroparalysis of the neuromuscular junction of insects and mammalians, acting much more rapidly on the insect than on the mammalian nerve terminals. This activity appears to be functional to the life style of the scorpion *Tityus serrulatus*, which relies on the rapid paralytic immobilization of the prey. The more rapid effect on the insect than on the mammalian preparation is consistent with an evolutionary driven adaptation to insect preys. The neuroparalytic effect of antarease appears to be dependent on a metalloprotease activity as the change of the Glu residue of its HExxH metalloprotease motif with Ala generates a mutant that does not display any neuroparalytic activity. These data are consistent with a previous report that antarease removes by proteolytic cleavage nearly the entire cytosolic domain of VAMP2 [16]. In fact, VAMP2 proteolysis by tetanus and by botulinum neurotoxin types B, D, F and G was long known to paralyze nerve terminals [20,30,31,32,33]. However, we were unable to detect any proteolytic activity of recombinant antarease versus VAMP2 using the same substrate and conditions used previously [16]. Such a discrepancy clearly requires further investigation.

However, some considerations can be made to contribute to the design of further experiments aimed at clarifying the mechanism of action of antarease and of the related scorpion metalloproteases [10]. The analysis of the primary structure of antarease clearly shows that it belongs to the family of metalloproteases called ‘metzincins’ because of a conserved methionine [19,24]. On the contrary, tetanus and botulinum neurotoxins and the anthrax lethal factor appear to form a unique metalloprotease family, characterized by a lack of conserved methionine and by the presence of a Tyr and an Arg residues within the second shell of zinc coordination [34,35,36,37,38]. In addition, the botulinum neurotoxins recognize VAMP2 via exosites, located at a distance from the active site [39,40], which are not present in the antarease sequence. 

The very rapid neuroparalytic action of antarease speaks against the possibility that this protein is internalized after binding and that it crosses an intracellular membrane to reach the cytosol where VAMP2 is located, as botulinum neurotoxins do [23]. In addition, it should be considered that the metalloprotease domain of tetanus and botulinum neurotoxins is capable of entering the cytosol of nerve terminals because it is assisted by a 50 kDa domain, termed HN, which is not associated with antarease. Hence, antarease by itself should not be capable of membrane translocation and of cytosolic metalloprotease activity.

On the basis of the results presented here, we favor an alternative hypothesis, which posits that antarease is a metalloprotease capable of binding and cleaving a membrane protein of the surface of the presynaptic membrane, which is thus inactivated. The nerve terminal target of antarease is a protein important for the release of neurotransmitter. The nature of this protein is unknown, but the paired-pulse facilitation induced by antarease, but not by its active site mutant, suggests that it might be a voltage-gated Ca^2+^ channel. In paired-pulses facilitation, the second augmented response has been interpreted as being due, in large part, to residual calcium loaded into pre-synaptic terminals during the first response, which has not been buffered or cleared prior the second response [41]. Considering the previous results on EJPs, these findings suggest that antarease causes a severely reduced release probability, being responsible with other toxins, for the toxicity of *Tityus* venom towards mammals and insects. However, as synaptic vesicle release can be substantially elevated under high-frequency stimulation, we conclude that the core fusion machinery is still operational, in accordance with our in vitro results, which show no cleavage of VAMP2, and that the reduced release is mainly due to defects upstream of the synaptic vesicles fusion process.

## 4. Materials and Methods

### 4.1. Animals

*Drosophila melanogaster* Canton S flies (Bloomington Stock Center, Bloomington, IN, USA) were raised in standard agar/yeast medium and kept at constant temperature (23 °C) and humidity (70%) under a 12-h light and 12-h dark photoperiod. 

CD1 mice (25–28 gr) were supplied by the Animal House of the Università degli Studi di Padova. All mice were housed and handled in accordance with the Ethical Committee of the University of Padua and experiments were authorized by the Italian Ministry of Health in date 11 May 2015 (authorization number 359/2015-PR). Mice were housed in a 12-h light–dark cycle with food and water access ad libitum.

### 4.2. Recombinant Antarease and Its Mutant E162A Cloning, Expression, and Purification

The cDNA sequences of antarease (UniProt Database, accession number P86392) or its active site mutant where Glu162 was substituted by Ala to abolish the metalloprotease activity [19] were codon optimized for expression in *E. coli* and synthesized by GeneArt. It was cloned into pRSET A (Invitrogen, Carlsbad, CA, USA) as a *BamHI*/*HindIII* insert with a *N*-terminal c-Myc (EQKLISEEDL) tag. The mutant E162A (numbered according the sequence reported Uniprot code P86392) was obtained using the same strategy, after the substitution of GAA into GCA to codify an alanine instead of glutamate. Both constructs were expressed in *E. coli* BL21 (DE3) strain induced with 1 mM isopropyl-β-d-thiogalactoside for 4 h. The purification protocol was adapted from Lacy and Stevens (1997) [42]. Briefly, the bacterial pellet was re-suspended in binding buffer 500 mM NaCl, 5 mM imidazole, 0.3 mM methionine, 20 mM Tris, and pH 7.9, in the presence of protease inhibitors EDTA-free (Roche, Indianapolis, IN, USA). Additionally, 0.5 M MgCl_2_ and 20 mg/mL DNase were added. Cells were lysed using a French press and the soluble and insoluble portion separated by centrifugation. The pellet was re-suspended by overnight stirring in binding buffer in the presence of 1% n-lauroyl sarcosine. Following centrifugation, the supernatant was purified using metal affinity chromatography HisTrap HP columns (GE Healthcare, Uppsala, Sweden) under manufacturer’s instructions. The pooled fractions were dialyzed in 250 mM NaCl, 20 mM Tris, pH 7.4 in the presence of 0.1% of n-lauroyl sarcosine. The protein identity was assayed by immunoblotting using His•Tag^®^ Monoclonal Antibody (EMD Millipore, Billerica, MA, USA) and Monoclonal Anti-c-Myc antibody produced in mouse clone 9E10 (Sigma-Aldrich, Saint Louis, MO, USA). 

The plasmid encoding for the full-length *Drosophila simulans* VAMP (termed nSyb) was kindly provided by Prof. Hiesinger. It was subcloned into pET-28a (Novagen, Gibbstown, NJ, USA) expression vector between EcoRI/XhoI restriction sites. The construct was then checked by DNA sequencing. *E. coli* BL21 (DE3) were induced with 1 mM IPTG, followed by overnight culture at 16 °C. Purification of nSyb was carried out by metal affinity chromatography as described above.

### 4.3. In Vitro VAMP2 Cleavage

1 μg of rat recombinant VAMP2 (1–97) expressed and purified as described previously [43] was incubated in 50 mM Hepes, 150 mM NaCl, pH 7.4, for 2 h at 37 °C under stirring with 1 μg of recombinant antarease or of its active site mutant E162A. 1 μg of BoNT/B was used as a positive control of VAMP2 cleavage as described [44]. Proteolysis were estimated by determining the decrease of intact VAMP2 with time. To ensure that the cleavage was due to the zinc endopeptidase activity of the toxin, the recombinant proteins were pre-incubated for 30 min at 37 °C in the presence of 10 mM EDTA and then the substrate was added. The assay of proteolysis of n-syb was carried out as described above in the presence of 0.1% octyl-glucoside to disperse nSyb.

### 4.4. Electrophysiology on NMJ of Third Instar Larva Preparation

Larval body wall preparations were dissected out in Ca^2+^-free HL3 solution from third instar larvae pinned on Sylgard coated petri dishes [45,46]. Central nervous system was excised by cutting segmental nerves roots.

After replacing Ca^2+^ free HL3 solution with Ca^2+^ 1 mM HL3, post-synaptic potentials at neuromuscular junction of fiber 6/7 of abdominal segments A3/A4 were intracellularly recorded, at room temperature (20–22 °C) in current-clamp condition, using an intracellular microelectrode (tip diameter 0.5 μm, 15 MΩ resistance). The recorded signal were amplified by a current-clamp amplifier (SEC 05, NPI, Tamm, Germany), digitized at 10 kHz sampling rate using an A/D interface (National Instruments, Austin, TX, USA) and fed to a computer for display and storage using an appropriate software (Win EDR, Strathclyde University, Glasgow, UK).

Fibers with a resting membrane potential below −60 mV were considered for the experiment. In these fibers, membrane potential was set at −70 mV throughout the experiment by injecting current through the intracellular electrode. Evoked post-synaptic potentials were recorded by stimulating at 0.1 Hz (pulse duration 0.4 ms; 1.5 threshold voltage) the segmental nerve using a suction electrode (tip diameter ~10 μm) connected to a stimulator (S88, Grass, Pleasanton, CA, USA) through a stimulus isolation unit (SIU5, Grass, Pleasanton, CA, USA). Paired-pulse facilitation was tested delivering paired-stimuli to the segmental nerve at 0.5 Hz with inter-stimulus intervals of 20, 50, and 100 ms. For each inter-stimulus interval tested, five consecutive paired stimuli were delivered.

Evoked post-synaptic potentials or paired-pulse facilitation were tested, in two separated sets of experiments, in the same fibre throughout an entire experiment, before, immediately after (t0) or at specific time points after adding to the bath recombinant antarease or its active site mutant or vehicle alone. During this period, intracellular microelectrode remained inserted in the fiber and resting membrane potential was continuously checked. In case of a reduction of membrane potential or an increase of current injection for maintaining membrane potential at -70 mV (both indications of a fiber membrane damage), the experiment was discarded. Intracellular recordings were analyzed offline using pClamp software (pClamp, Axon, Sunnyvale, CA, USA). 

The relative amplitude of evoked post-synaptic potentials during the time course of each experiment, was calculated with respect to the amplitude of the first recorded evoked post-synaptic potential. Paired-pulse facilitation was calculated by the ratio between the second response amplitude and the first response amplitude. Statistical comparisons and graphs were made using Graphpad software (Graphpad, La Jolla, CA, USA) or MATLAB (Matworks, Natick, MA, USA).

### 4.5. Assay of the Mouse Hemidiaphragm Paralysis

Mouse phrenic nerve-hemidiaphragms were dissociated from male Swiss-Webster CD1 mice weighing about 20 g and mounted in 2–4 mL of oxygenated (95% O_2_, 5% CO_2_) Krebs-Ringer solution (137 mM NaCl, 5 mM KCl, 1.8 mM CaCl_2_, 1.0 mM MgCl_2_, 24 mM NaHCO_3_, 1 mM NaH_2_PO_4_, and 11 mM glucose, pH 7.4) at 37 °C. Two innervated hemidiaphragm preparations were isolated from each animal. Muscles were stretched to the optimal length for twitch response and the phrenic nerve was stimulated via two ring platinum electrodes with supramaximal stimuli of 10 V amplitude and 0.1 ms pulse duration with a frequency of 0.1 Hz. Muscle contraction was monitored with an isometric transducer (Harvard Apparatus, Massachusetts, USA); data were recorded and analyzed via an i-WORX 118 system with Labscribe software (Harvard Apparatus, Massachusetts, USA). The isolated nerve-muscle was allowed to stabilize for at least 10 min before recombinant antarease, or its active site mutant (final concentration 80 nM) or vehicle were added to the tissue bath. Muscular twitch was monitored until complete paralysis. Data were expressed as time to decrease the twitch to 50% of the initial value (paralysis half-time).

## Supplementary Materials

The following are available online at www.mdpi.com/2072-6651/9/3/81/s1, Figure S1: Paired pulse facilitation. Example of paired pulse facilitation following paired stimulation (black arrows) of segmental nerve innervating fibre 6 of abdominal segment 3 in third instar larva body wall preparation. The second Excitatory postsynaptic potential (EPP) is augmented with respect to the first EPP. Interstimulus interval is 75 ms (gray double arrow). Calibration bar on the right side of the figure, Figure S2: Purification of recombinant antarease. *T. serrulatus* E162A antarease was produced as recombinant protein in *E. coli* and subjected to SDS-PAGE. Lane 1: molecular weight markers; lane 2: total protein extract; lanes 3 and 4: washes; lanes 5: surnatant after sarcoside 0.5%; lanes 6-9: eluted fractions from the affinity chromatography containing E162A antarease (arrow), Figure S3: EJP Amplitude 500 s after addition of recombinant proteins. Mean ± Standard Deviation of EJP Amplitudes of 4 experiments for each experimental condition, measured 500 s after addition of recombinant proteins or vehicle to the bath. EJP amplitudes are relative to the mean of the last 10 EJPs recorded, before adding recombinant proteins or vehicle to the bath, in the same third instar *D. melanogaster* larval muscle 6/7 of A3/A4 segment. Microlectrode remained intracellularly placed throughout the entire experiment, Figure S4: P2/P1 ratio of double pulse stimulation. Mean ± Standard Deviation of Pulse 2/ Pulse 1 (P2/P1) of 4 experiments for each experimental condition, measured 650 s after addition of recombinant proteins or vehicle to the bath, when Antaerase was fully effective.
