# Supplementary material for: Electrophysiological Characterization of the Antarease Metalloprotease from Tityus serrulatus Venom
Source: Toxins (Basel). 2017 Feb 27;9(3):81. doi: 10.3390/toxins9030081 (PMC5371836; doi:10.3390/toxins9030081)
Supplement: Supplementary file 1 [file toxins-09-00081-s001.pdf]

Supplementary Materials: Electrophysiological Characterization of the Antarease Metalloprotease from *Tityus serrulatus* Venom

Irene Zornetta, Michele Scorzeto, Pablo Victor Mendes Dos Reis, Maria E. De Lima, Cesare Montecucco, Aram Megighian, and Ornella Rossetto

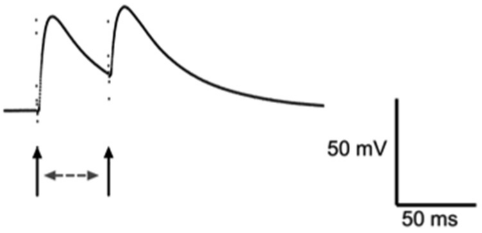

Figure S1. Paired pulse facilitation. Example of paired pulse facilitation following paired stimulation (black arrows) of segmental nerve innervating fibre 6 of abdominal segment 3 in third instar larva body wall preparation. The second Excitatory postsynaptic potential (EPP) is augmented with respect to the first EPP. Interstimulus interval is 75 ms (gray double arrow). Calibration bar on the right side of the figure.

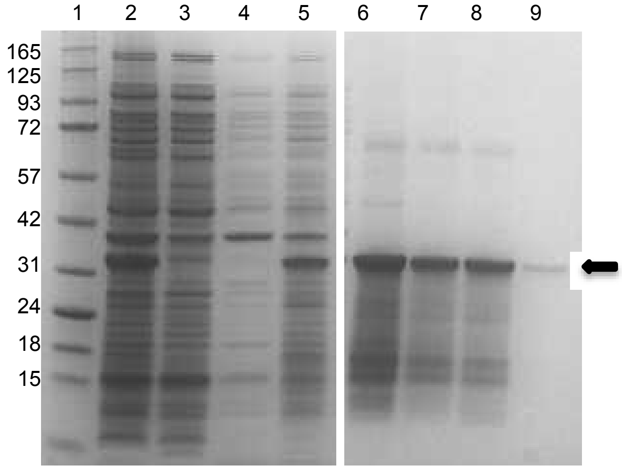

Figure S2. Purification of recombinant antarease. *T. serrulatus* E162A antarease was produced as recombinant protein in *E. coli* and subjected to SDS-PAGE. Lane 1: molecular weight markers; lane 2: total protein extract; lanes 3 and 4: washes; lanes 5: surnatant after sarcoside 0.5%; lanes 6-9: eluted fractions from the affinity chromatography containing E162A antarease (arrow).

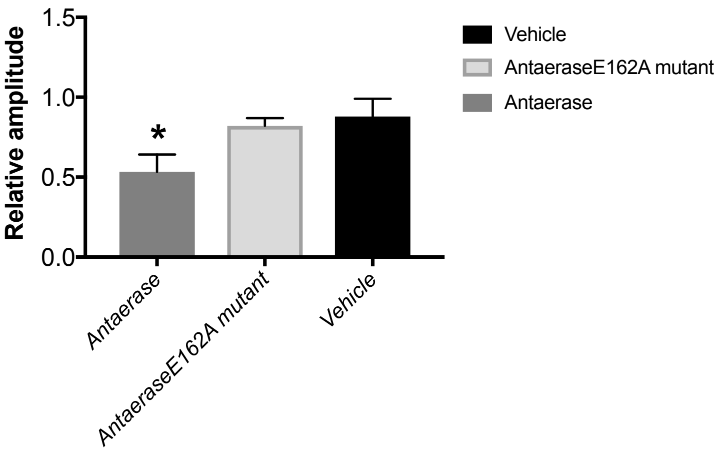

Figure S3. EJP Amplitude 500 s after addition of recombinant proteins. Mean ± Standard Deviation of EJP Amplitudes of 4 experiments for each experimental condition, measured 500 s after addition of recombinant proteins or vehicle to the bath. EJP amplitudes are relative to the mean of the last 10 EJPs recorded, before adding recombinant proteins or vehicle to the bath, in the same third instar *D. melanogaster* larval muscle 6/7 of A3/A4 segment. Microlectrode remained intracellularly placed throughout the entire experiment.

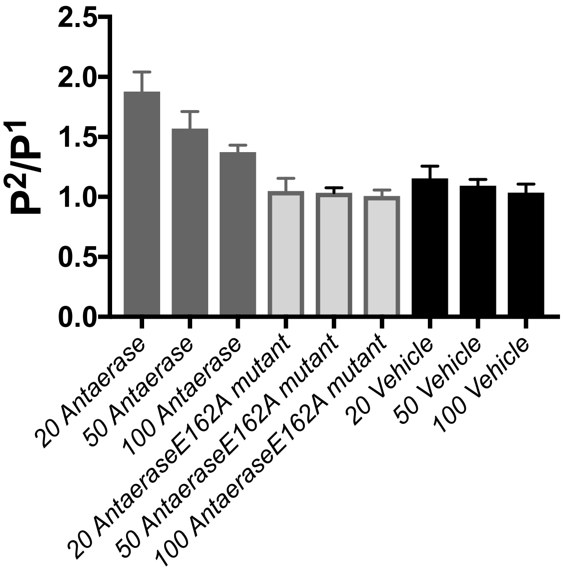

Figure S4. P2/P1 ratio of double pulse stimulation. Mean ± Standard Deviation of Pulse 2/ Pulse 1 (P2/P1) of 4 experiments for each experimental condition, measured 650 s after addition of recombinant proteins or vehicle to the bath, when Antaerase was fully effective.
